# Psychiatry past and present: do we need history?

**DOI:** 10.1192/bjb.2018.102

**Published:** 2019-06

**Authors:** Claire Hilton

**Affiliations:** 1 Historian in Residence, Royal College of Psychiatrists, UK

**Keywords:** History of psychiatry, National Health Service, policy, service development, quality improvement

## Abstract

Historical evidence can be useful to inform debate about current dilemmas in health service policy. However, concepts of historical analysis may be problematic for doctors, for whom a model of ‘history’ is often based on clinical history-taking: a clinical history aims to explain the present, whereas a historical analysis aims to elucidate the past. This article discusses and illustrates these concepts, and highlights potential pitfalls of poor historical methodology. It also provides pointers about researching the history of psychiatry in the UK and how to contribute historical evidence to health service policy debates today.

**Declaration of interest:**

None.

Recent media focus on the 70th anniversary of the National Health Service (NHS) is welcome. Each decennial anniversary has brought similar commemorative events, although in between, the NHS often forgets or ignores its history. The NHS tends to use recent studies from fields such as epidemiology, sociology or economics to help construct policies and plans, but has sometimes ignored evidence, using a ‘foundation of sand’^1^ instead. It rarely turns to historical evidence, which might provide a complementary and longer-term perspective, such as about the complex configuration of factors that underpin policy, including politics, economic circumstances, technical innovations, patient expectations and diverse motivations to change or maintain the *status quo*. For those involved in making health policy decisions at any particular time, it may be difficult to achieve a fully informed ‘helicopter view’, to weigh up how and why a multiplicity of factors come together. Historical analyses can provide helicopter views of events in the past, and trace developments over a period of time including into the very recent past. These may reveal influences on decision-making, which can contribute insights into today's dilemmas, raise questions about current policy options, stimulate imaginative consideration about alternative futures and formulate steps to achieve the best option.

NHS policies and plans are introduced when change is desired, so proposals appropriately focus on the future. The future is complex and challenging, but plans are presented as straightforward, upbeat and original, ‘a fresh mindset for mental health within the NHS and beyond’,^2^ with the message that we can achieve what has not been considered or done before. History does not repeat itself, as past events are contextualised by the period in which they happened, but behaviours and patterns of response may repeat, and societal beliefs, attitudes and values related to health and social care may change relatively slowly. Thus, including these as part of a multifaceted historical analysis can help shed light on past and present policies and practices. For example, why does ambivalence toward people who are mentally unwell, elderly, less able or otherwise stigmatised perpetuate, at least in modified forms?^3^ This cannot be answered simply, but subjective values and stereotypical attitudes may be associated with recurring discriminatory policies and under-resourcing of mental health and social care services.^4^ One outcome of this may be the increasing gap in parity of esteem between psychiatry and its physical illness counterparts.^5^

This paper aims to explore how and why doctors and other health service staff involved in NHS planning, policy and service development need to incorporate historical evidence into today's debates, and enable them to begin to do so effectively.

## What is history? How clinicians and historians understand it

When clinicians consider the past, they model their understanding on two practices drawn from their professional training: clinical history-taking and scientific literature reviews. Clinical history-taking gathers information from the past to understand a patient's illness in the present to plan future treatment. A scientific literature review is undertaken to justify a future scientific or clinical research proposal. It judges the past using the ‘retrospectoscope’ or hindsight, comparing it directly with today's standards and expectations. With a retrospective or hindsight analysis, it is easy to conclude that we can do better.

A historian, by contrast, aims to understand the *past* in its own context, not directly related to present or future objectives or through the eye of hindsight. For example, the Mental Treatment Act 1930 (England and Wales) can be used to demonstrate how a hindsight view can be disparaging, in contrast to a contextualised historical view, which indicates its humane motivation. The Act aimed to lessen stigma by abolishing the term ‘asylum’, replacing it with ‘mental hospital’, bringing it into line with the term ‘hospital’ for physical illness. It also introduced ‘voluntary’ admission to mental hospitals. Voluntary patients, however, had to give 72 h notice in writing if they wished to be discharged. A hindsight analysis, voiced to me by several clinicians recently, concludes that the patient was, by definition, not ‘voluntary’. However, a historical view, seeking to understand the Act in the context of the time, provides an alternative perspective. Most families had neither a telephone nor a car, and a mental hospital could be located remotely from centres of population many miles from their, and the patient's, home. Seventy-two hours allowed the hospital to send a letter to a patient's relative or friend, who could then arrange to take a day off of work to travel to the hospital, with the aim of providing support for the patient immediately after discharge.

Hindsight methods are largely linear interpretations of events, and may be described as ‘Whiggish’ or ‘Whig History’.^6^ The term is often applied pejoratively to histories that present the past as an inevitable march of progress toward enlightenment. Whig history puts faith in the power of human reason to reshape society for the better, which implies that we are where we are now and need only build on the last step. It does not encourage deeper exploration, which usually reveals that real-world events are multifactorial webs and far from linear. John Turner argued the need to abandon ‘single-issue mythologies’ around the history of mental health service provision,^7^ and to explore the numerous interacting factors that influence it (Box 1).

Box 1 Some of the overlapping and interacting factors that influence mental health service development.

• Values, stereotypes and understanding of mental illness, e.g.
  the media
  priorities and balance of:
    deserving and undeserving, notions of the Victorian Poor Law
    general hospitals and community services
    high-tech and low-tech medicine
    risk, safety and danger
• National factors, e.g.
  costs
  desire to achieve outcomes within the duration of a single parliamentary term
  economic policies
  ideology
  law
  politics
  proximity to elections
  regulation
  risks
  scandals and maintaining appearances
• Local factors, e.g.
  interpretation of national guidelines
  priorities of commissioners, NHS trusts or others who control the money-flow
  recruitment and retention
• Personal experience of patients, families and staff, e.g.
  illness, treatment and outcome
  patient–practitioner relationships
  tolerance of poor services by patients, families and staff, who may campaign for better, or fearful of stigma or losing services or jobs, do not complain or whistle-blow

Present day concerns may trigger the desire of healthcare staff and historians to understand the past, and both groups need to ensure objectivity to minimise the risks of subjective interference by contemporary experiences. Medical-style analyses of the past usually lead to a reductionist conclusion, either a diagnosis or a research question. In contrast, historians provide an expansive, contextualised conclusion. If historians then want to relate past to present to inform current debate, they will undertake an additional analysis, as exemplified in the peer-reviewed journal *History and Policy* (http://www.historyandpolicy.org/).

## How, and how not, to do history: some examples

To analyse historically, we must travel back in time, to look around the environment we seek to understand, and to criticise, explain and interpret it from within. To compare hindsight and historical methods, we can take as an example, Ronald ‘Sam’ Robinson's (1924–2014) description of his induction as a junior hospital doctor at Crichton Royal Hospital, Dumfries, in the early 1950s. Crichton Royal was a centre of excellence for research in psychiatry. Willy Mayer-Gross, formerly Professor of Psychiatry at Heidelberg, was Director of Research. Robinson did not recall being given any reason for this part of his induction, but he may have been aware that using drugs to induce an ‘experimental psychosis’ was a recognised method at that time for clinicians aiming to gain a greater understanding of psychotic phenomena.^8^ Robinson wrote:

‘Mayer-Gross was a warm ebullient pyknic with a sparkling eye. A week or two after my arrival he invited me to come to his office at nine o'clock the following morning. My colleagues warned me that this would be for my statutory dose of LSD [Lysergic acid diethylamide] - and so it turned out; no ifs or buts. After the colourless and tasteless drink my reactions and sensations were monitored for the next four hours by M-G [and others]. Among the various procedures was an EEG [electroencephalogram]. My peers had regaled me the previous evening with expectations of vivid visual and tactile hallucinations, pictures sliding down walls and multiple delusions. To my disappointment none of these occurred; it was for me a complete non-event.’ (Robinson, 2009: pp. 91-92)^9^

Using hindsight methodology, it is easy to criticise this experimentation as unethical, and congratulate ourselves that we do inductions and research better. A historian, however, would probably ask: Why did they do it that way? What were the alternatives? What were the General Medical Council guidelines? How did it fit with the Nuremberg Code or other research ethics? What can it teach us about research processes at the time? Only then would a historian attempt to answer the question of what it may teach us for today's practice.

Malaria inoculation, a dangerous treatment for general paralysis of the insane (arising from neurosyphilis), provides another example to compare hindsight and historical interpretations. Malaria inoculation induced high temperatures, which could kill spirochaetes and therefore cure syphilis, but it could also kill the patient. Today, with antibiotics available, malaria inoculation would be unethical, but when Julius Wagner-Jauregg introduced it in Austria in 1917 (and subsequently won the Nobel Prize for it), it changed an inevitably terminal illness with death commonly taking place in an asylum, into a potentially treatable condition. It would be hard to criticise the use of malaria inoculation in the context of psychiatric practice a century ago. If anything, we could criticise the delay in introducing it to the UK: over 1000 (1% of total) in-patients in English mental hospitals suffered from general paralysis of the insane, but in 1922 only eight were treated, and in 1929, only one-third of mental hospitals used the technique.^10^^,^^11^ Why, one might ask, was it so slow to be adopted? What caused the delay? Did the same happen in other countries? A retrospective, hindsight analysis discredits the treatment; a historical analysis explores the context of policies, practices, values, attitudes and choices at the time.

The story of psychiatrist Russell Barton (1923–2002) also highlights some of the risks of using hindsight to analyse the past, and of directly comparing past and present. Barton was one of 96 London medical student volunteers who went to Belsen concentration camp in Germany to help survivors, 2 weeks after its liberation by the British Army in 1945. His experience there inspired his passion and persistence to provide humane and dignified treatment and care for patients in psychiatric hospitals. In 1968, he wrote, in a widely read history magazine, about the medical students’ experiences and his understanding of Belsen based on his time there.^12^ He directly compared the authorities controlling Belsen in 1945 with those managing NHS psychiatric hospitals in the 1960s. He noted similar harmful psychological consequences for those held within, and commented that the public appeared to turn a blind eye to inhumanities taking place on their doorstep, whether in Germany during the war or in England in the 1960s. Some of his interpretations of Belsen were incorrect, and his comparisons between Belsen and psychiatric hospitals outraged readers, who articulated their thoughts in the national press. Subsequently, Holocaust deniers latched onto his article. An internet search about Barton today finds him mainly on Holocaust denial websites, rather than where he deserves to be, which is remembered as a psychiatric reformer and innovator.^13^

Historian Andrew Scull highlighted some perils of doctors undertaking historical research. In 1991, he described psychiatrist-historians as a ‘peculiar group of amateurs’ whose:

‘…distortions have seriously compromised the scholarly usefulness of the accounts offered - creating versions of the past that serve (in ways generally obscured from their authors) to legitimate the profession's present-day activities; or that represent a harmless form of antiquarianism but largely fail to satisfy the elementary canons of good historiography.’ (Scull, 1991: p. 239)^14^

Recently, Scull admitted that clinician-historians who combine ‘psychiatric expertise and serious historical scholarship’ have ‘greatly enriched the sophistication and the range of questions that have come to mark work in the field.’^15^ Collaborative work between historians and psychiatrists may help ensure that clinicians use ‘serious historical’ methodology, and may help historians better understand and interpret aspects of medical terminology and clinical practice.^16^

## Starting a historical study

Understanding the differing concepts of ‘history’ in clinical practice and historical analysis is crucial to undertaking good historical research. In 2016, Wendy Burn, then Dean of the Royal College of Psychiatrists, agreed that higher trainees in psychiatry could undertake a historical study as part of their research portfolio, provided the methodology was appropriate. Some useful and easily accessible sources, with a focus on policy and development of psychiatry in the UK over the past two centuries, are listed in Box 2.

Box 2 Where to start: primary sources for history of psychiatry in the UK.

Journals:

*British Journal of Psychiatry*
*BMJ*
*The Lancet*
*Hansard*

Books:

textbooks (compiled at the time)
novels, e.g. Charles Reade, *Hard Cash* (1865); Phyllis Bottome, *Hidden Worlds* (1934)
The British Library
The Wellcome Library (including online resources, and archives)

Archives:

medical: Royal Colleges
local: county records offices
national:
  The National Archives (UK Government and England and Wales: www.nationalarchives.gov.uk/)
  National Records of Scotland (https://www.nrscotland.gov.uk/)

Oral history:

Oral History Society (http://www.ohs.org.uk/)

Other:

Andrew Roberts, Mental Health History Timeline (http://studymore.org.uk/mhhtim.htm)
History of Psychiatry Special Interest Group, Royal College of Psychiatrists resources (https://www.rcpsych.ac.uk/members/special-interest-groups/history-of-psychiatry/resources)

## History and now

There are several current national policy issues in psychiatry for which historical analysis may provide insight and stimulate discussion. These include quality improvement, deprivation of liberty safeguards and parity of esteem.^17^ Space permits exploration of only one here.

### Quality improvement

Today, quality improvement initiatives aim to create, improve, monitor and adjust services to meet patients’ needs in a local context. Quality improvement encourages low hierarchical, engaged clinical teams who are in daily touch with patients, to think creatively and innovatively to ensure delivery of evidence-based clinical practice. Recently introduced to the UK from the Institute for Healthcare Improvement in the USA, quality improvement is regarded as new.^18^

A historical study in the UK, however, shows that psychiatrists used almost identical methods between the 1950s and 1970s. At Claybury Hospital, for example, it enabled staff and patients together to create therapeutic community-type wards to promote rehabilitation and discharge. Change achieved incrementally in clinical practice was then disseminated.^19^ Similar methods were used to develop proactive, therapeutic old-age psychiatry services comprising domiciliary assessment, out-patient clinics, day hospitals and short-stay wards, making many long-stay wards redundant.^20^^,^^21^ Clinical leaders inspired these developments, intuitively adopting quality improvement-type methods. They worked in multidisciplinary teams with a low hierarchical structure, and opinions of front-line staff helped shape services. Staff took ‘ownership’ of the new patient-focused services, and improvements in staff morale and recruitment were noted.

If a successful quality improvement-like model existed in the UK in the middle of the 20th century, questions arise about how and why it ceased to function. The explanation may hinge on the balance of clinician and manager roles in the NHS leadership equation. Greater prominence of managerial ranks since the 1970s was associated with more directive top-down policies and uniformity. Admirably, this sought to achieve minimum standards everywhere. However, it also diminished a workplace culture of staff involvement, innovation, ownership and incentive to achieve above the minimum. Knowledge of the past thus identifies matters that need to be discussed today and, specifically for quality improvement, if it is successful, how to promote and perpetuate the culture of bottom-up innovation and prevent it from being abandoned again.

## Comment

The examples used in this paper may be criticised as overly anecdotal. However, doctors typically learn from their experiences of individual patients as ‘case studies’, and these illustrations aim to serve a similar purpose. Personal experience of historical research and discussion with psychiatrists, such as in the Royal College of Psychiatrists History of Psychiatry Special Interest Group, suggests they provide meaningful and broadly representative examples.

Correct methodology is vital for historical analysis, just as it is for good clinical practice and scientific research. High-quality historical data are important to inform policy and NHS development, in a similar way to clinical research evidence informing work with individual patients. However, both need to be ‘translated’ into practice through the appropriate route. Academic departments of history of medicine and psychiatry are flourishing, such as the Centre for the History of the Emotions at Queen Mary University of London. Relevant historical analyses are being published, thus using high-quality historical evidence in policy debate is becoming more feasible, even if one does not undertake the historical research personally.

In summary, doctors can despair at, and ridicule, some self-publicising amateurs as ‘quacks’. Historians like Scull may do the same, and ridicule medical historians using inappropriate methodology as whiggish (or worse). Assuming that today's plans and innovations are entirely new and lack any historical precedent precludes learning from past experience. Historical evidence may be less tangible than data habitually used for planning and changing health services, but ignoring it is perilous.

## About the author

**Claire Hilton** is Historian in Residence, Royal College of Psychiatrists and Research Affiliate, Centre for the History of the Emotions, Queen Mary University of London, UK
